# A Metabolomics Approach to Pharmacotherapy Personalization

**DOI:** 10.3390/jpm8030028

**Published:** 2018-09-05

**Authors:** Elena E. Balashova, Dmitry L. Maslov, Petr G. Lokhov

**Affiliations:** Institute of Biomedical Chemistry, Pogodinskaya St. 10, Moscow 119121, Russia; dlmaslov@mail.ru (D.L.M.); lokhovpg@rambler.ru (P.G.L.)

**Keywords:** pharmacometabolomics, metabolomics, pharmacogenomics, therapeutic drug monitoring, personalized medicine, mass spectrometry

## Abstract

The optimization of drug therapy according to the personal characteristics of patients is a perspective direction in modern medicine. One of the possible ways to achieve such personalization is through the application of “omics” technologies, including current, promising metabolomics methods. This review demonstrates that the analysis of pre-dose metabolite biofluid profiles allows clinicians to predict the effectiveness of a selected drug treatment for a given individual. In the review, it is also shown that the monitoring of post-dose metabolite profiles could allow clinicians to evaluate drug efficiency, the reaction of the host to the treatment, and the outcome of the therapy. A comparative description of pharmacotherapy personalization (pharmacogenomics, pharmacoproteomics, and therapeutic drug monitoring) and personalization based on the analysis of metabolite profiles for biofluids (pharmacometabolomics) is also provided.

## 1. Introduction

The uniformity of the drug response or low inter-individual differences in drug response are commonly accepted tenets in the field of medicine. Almost all drugs are prescribed on the basis of this statement. This approach can be described as treatment of the “average patient” by “the average pill” or “one size fits all”.

**Figure 1:**
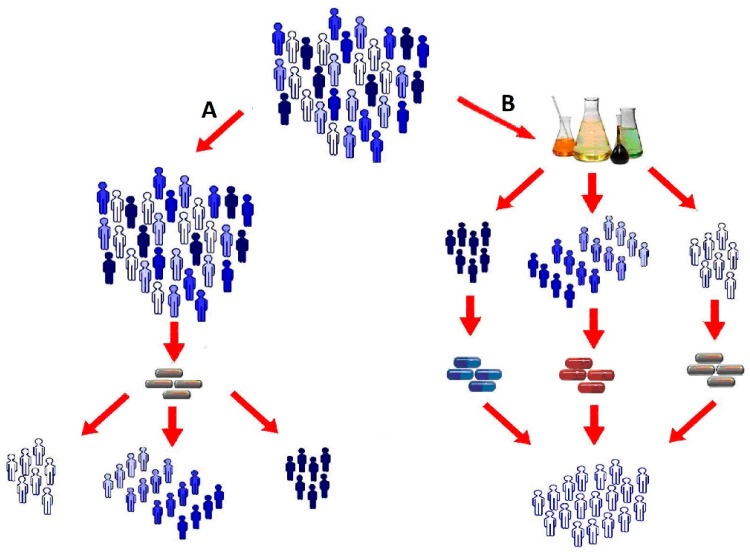
A schematic of the traditional (arrow **A**) and individual (arrow **B**) approaches to medical treatment.

However, clinicians have long observed that the actual effectiveness of the pharmacotherapy may be variable. A medication may be ineffective for a significant proportion of patients or provoke the development of adverse drug reactions (ADR) (Figure 1A). According to published data, current pharmaceutical treatments are not effective for 30–60% of patients [1,2,3], and the percentage of patients developing ADR can be as high as 30% [4]. The reason for this individual variation can be related to: genetic polymorphisms, individual reactions (from tolerance to increased sensitivity to the drug), age, gender, addictions (smoking, alcohol, etc.), race and ethnicity factors, drug-related factors (multiple drug therapy, distribution of generics, side effects not detected in clinical trials), the impact of environmental factors, accompanying diseases, etc. [5]. All these factors point to the necessity of selecting a therapy according to patients’ individual characteristics [6] (Figure 1B).

The individualized plan of medical treatment aims to maximize drug effectiveness and to reduce the side effects. This approach is called “personalized medicine” [7]. The idea of personalizing treatment (“tailoring medical treatment”) is not new. The personalization of treatment based on available individual information has long been used by physicians. Known characteristics of the individual (age, weight, etc.), as well as clinical and laboratory parameters (co-morbidity, family history, biochemical parameters) can be used to customize drug treatment [8]. For example, a significant rise in the levels of urea and ornithine (a urea cycle intermediate) in the blood with age could reflect either an increase in hepatic ureagenesis or a decrease in the rate of urea clearance by the kidney [9]. For individuals of some ethnicities, the increase of the body mass index during aging is associated with increased levels of the xenobiotic paraxanthine. Levels of other xenobiotics, including caffeine, are also reported to increase in older subjects, possibly reflecting decreased activity of hepatic cytochrome P450 (CYP) enzymes [9]. However, rapid developments in the fields of genetics, molecular biology, and biochemistry have allowed the concept of personalized medicine to evolve greatly.

New analytical approaches, mainly developed over the last two decades, allow for a comprehensive analysis of the organism at any level of organization, from genes to low-molecular-weight substances [10,11]. These methods are related to genomics, transcriptomics, proteomics, or metabolomics (“omics” sciences) and are used for the investigation of the genome, transcriptome, proteome, and metabolome, respectively.

**Figure 2:**
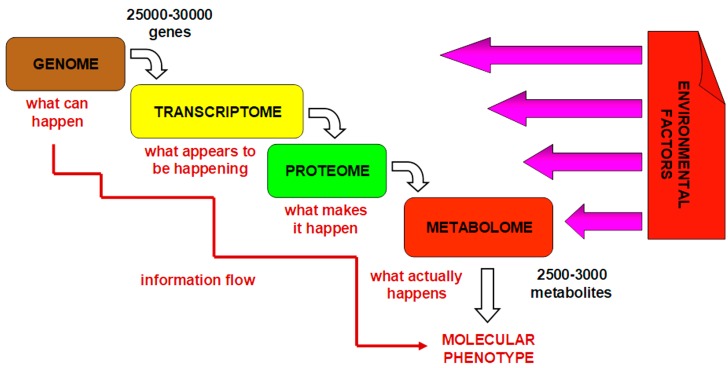
A biological system represented as a complex interaction of the genome, transcriptome, proteome, and metabolome.

Metabolomics is the analysis of metabolomes present in biological samples at a defined time point and under a specific set of conditions [12,13]. The term “metabolome” refers to collection of low-molecular-weight substances (<1000 Da). These substances (metabolites) are final and intermediate products of metabolism. Figure 2 shows that the metabolome is a final downstream product of other “omes”. Thus, the metabolome is a result of complex interactions among gene expression, protein expression, and the environment (including lifestyle, diet, gut, disease processes, drug therapy, etc.) [14,15]. Therefore, the metabolome is the integrative and most informative level that provides a complete overview of an organism’s current physiologic status in real time.

**Figure 3:**
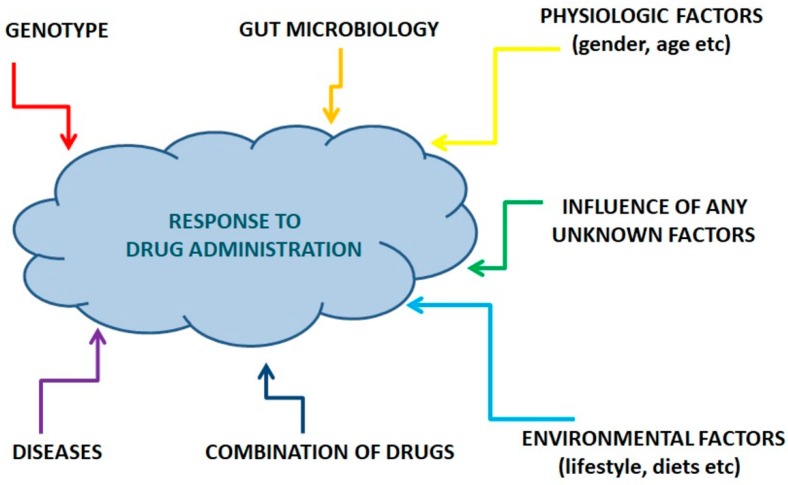
Factors that determine individual drug responses.

The youngest and most promising practical branch of metabolomics is pharmacometabolomics (PhM), also known as pharmacometabonomics, which was described previously in the context of pharmacotherapy personalization [14]. Pharmacometabolomics applies a metabolomic approach to the research of drug effects on individuals to understand the mechanism of action (pharmacodynamics) and metabolism (pharmacokinetics) of the drug, detect the factors that can alter drug metabolism, identify biomarkers relevant to the response of an organism to drug administration, etc. [16]. The analysis of this information allows clinicians to predict the effectiveness and toxicity of a proposed drug therapy for an individual [17,18]. Metabolome monitoring during drug therapy increases its effectiveness, allowing the monitoring of the reactions of the body caused by both the introduction of the pharmacological substance and the body’s response to various external factors (intestinal microflora, environmental factors, etc.) [19,20] (Figure 3).

At the present time, most researchers consider PhM as a method for predicting drug effectiveness prior to dosing. However, the possibilities of PhM for the personalization of treatment are much broader. Monitoring the post-dosing metabolite profile is also very important for the individualization of therapy. A comparison of metabolite profiles before and after administration of the drug allows the clinician to: register changes in the concentration of metabolites, identify metabolic processes that were altered by the drug administration, identify and assess the impact of metabolites that appeared during the current therapy, thus providing information about the rate of drug metabolism and drug effectiveness and toxicity. This information allows the researcher to estimate the effectiveness of the current medication as well as the disease status. The therapeutic regimen can be modified on the basis of the data obtained [7,19,20]. Such an approach to the personalization of treatment would be truly comprehensive. It would be possible to select the most effective drug prior to treatment, as well as to correct the therapy in accordance with the individual’s characteristics (tailoring the treatment).

In this review, we explore the challenges and opportunities of PhM as a potential platform for personalized medicine. Pharmacometabolomics was compared with two other “omics” technologies, pharmacogenomics (PhG) and pharmacoproteomics (PhP), which are also suitable for drug treatment customization, and with therapeutic drug monitoring (TDM), which is currently the most widespread method for the individual adjustment of drug dose [21].

## 2. Pharmacometabolomics Methodology

Typically, the first step of a metabolomics study is the collection of the samples of interest (e.g., plasma, cerebral spinal fluid, biopsied tissue). Low-molecular-weight substances (metabolites) are extracted, separated, and quantified.

Metabolomics methods fall into two distinct categories: targeted metabolomics (the measurement of defined, chemically characterized, and biochemically annotated metabolites) and untargeted metabolomics (a comprehensive analysis of all measurable metabolites in a sample, including unknown substances) [13,22]. Metabolic profiling relates to the second group. The metabolite profile represents all interactions among various aspects of the genome, microbiome (symbiotic human flora), parasites, and the environment. Therefore, a metabolite profile is very informative not only regarding the dose of a given drug, but also for modifying pharmacotherapy on the basis of a patient’s characteristics, including genetic variation, metabolism, and environmental intervention [14,19,23]. Using metabolic profiling, PhM attempts to quantify drug interventions depending on a pre-dose mathematical model of an individual’s metabolic status.

Today, nuclear magnetic resonance (NMR) spectroscopy and mass spectrometry (MS) are the methods used most frequently for biofluid metabolic profiling [24,25,26]. Table 1 summarizes the main advantages and disadvantages of metabolomics analytical techniques [27,28,29,30,31,32,33,34,35,36,37,38]. Nuclear magnetic resonance spectroscopy is a quantitative and highly reproducible method. The primary disadvantage of NMR spectroscopy compared to other analytical platforms is its low sensitivity. Nuclear magnetic resonance spectroscopy can reliably detect and quantify only metabolites present at relatively high concentrations. Using simple (i.e., unenhanced by chromatography) NMR, only several tens of metabolites can usually be detected in blood plasma or serum [39].

The MS method employs a unique combination of good selectivity and sensitivity (to the femtomole level) [40,41]. Mass spectrometry is a highly sensitive technique that is capable of detecting a diversity of metabolites with high precision. The MS method can be categorized as direct mass spectrometry and mass spectrometry, which are utilized in combination with separation techniques: high-performance liquid chromatography (LC–MS) or gas chromatography (GC–MS). The combination of mass spectrometry with various separation techniques simplifies the identification of metabolites and is used frequently in scientific studies. Unfortunately, several limitations prevent the widespread use of these approaches in the clinic. The two main limitations are the long time required for analysis and the diversity of the separation equipment components (chromatographs, columns, etc.) and separation protocols (eluents, gradients, etc.). Applying different components and protocols in chromatography limits the method’s reproducibility and leads to the collection of false data. Therefore, method standardization is one of the most important requirements in the application of MS-based methods in clinical practice.

**Figure 4:**
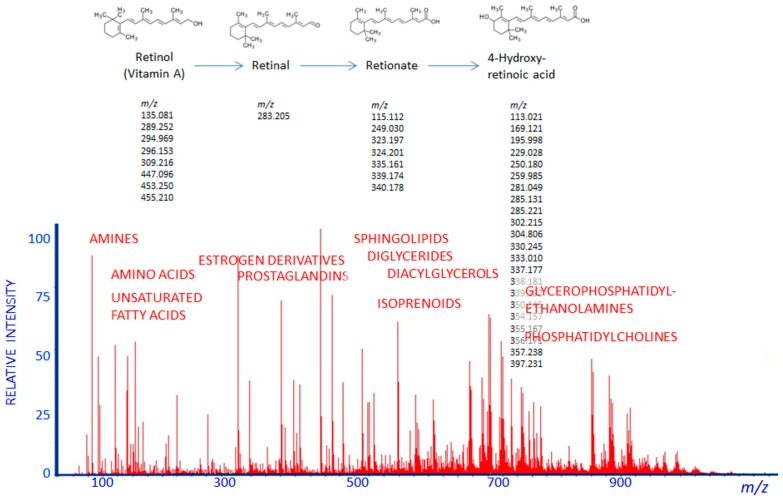
Mass spectrum of human blood plasma metabolites acquired by DIMS in the positive mode of micrOTOF-Q (BrukerDaltonik Ltd., Billerica, MA, USA). The labels indicate the different metabolite groups detected. The upper panel is a representative image of biodegradation (in this case, of vitamin A). The *m*/*z* values for mass peaks of vitamin A and its derivates (adducts, fragments, and multi-ions) are also presented.

Direct-flow infusion mass spectrometry (DIMS) is one possible application of metabolite analysis in clinical practice [42,43,44,45,46,47,48]. Direct-flow infusion mass spectrometry allows biological materials to be applied directly to the ionization source of a mass spectrometer without any preliminary separation steps [49,50]. An absence of prior metabolite separation dramatically simplifies the analysis, thereby reducing the time of execution and increasing results’ reproducibility. The results of metabolic profiling using plasma samples from kidney cancer patients and healthy volunteers analyzed by DIMS and LC–MS showed that the two methods were similarly effective. While DIMS and LC–MS exhibit commensurate classification and prediction capabilities, DIMS consumes only 5% of the analysis time required for LC–MS. The lack of effective metabolite separation is compensated for by using modern mass spectrometers with high mass resolution, which can detect most metabolites at physiological concentrations (Figure 4). The effectiveness of this approach for metabolic profiling has been confirmed in a series of experiments [42,43,47,48,51], and it appears to be a method that can be optimized for metabolomics-based clinical tests. The high efficiency of DIMS was demonstrated in the metabolomic diagnosis of prostate cancer and lung cancer patients, as well as in the diagnosis of impaired glucose tolerance using mass spectrometers equipped with electrospray ionization (ESI) ion sources [42,47,52]. Additionally, DIMS provides an opportunity to compare mass spectrometric metabolite profiles obtained for the same type of sample in different laboratories. This is a key requirement for implementing any omics-based testing in the clinic [52].

After the analysis is complete, the resulting datasets must be further processed; this process may be time-consuming [26]. Data are analyzed with one or more software packages designed for use with large datasets. A database is then created for each individual, to compare measurements obtained before and after the drug therapy. These databases include information pertaining to the levels of detectable metabolites as well as the identity and characteristics of metabolites (e.g., oxidation–reduction potential, mass/charge ratio). Software tools may then be used to: (a) identify disease signatures (e.g., compounds that indicate a disease state) (b) identify unrecognized groups in the data (e.g., subgroups based on drug response); (c) identify interactions between variables; (d) map variables to known biochemical pathways.

The datasets are analyzed using a series of high data density informatics approaches. The informatics outputs shown here include class prediction (SIMCA-P, Umetrics, Umeå, Sweden), principal component analysis of a computationally modeled dataset (SIMCA-P, Umetrics), 2D cluster analysis (GeneLinker Platinum, Improved Outcomes Software, Kingston, ON, Canada), metabolic analysis (http://www.biotech.icmb.utexas.edu), and cluster analysis (from Piroutte, Infometrix, Bothell, WA, USA) (for a review, see reference [30]).

The selection of analytical equipment and software is often determined by the purposes of the research; each type of tool has unique strengths and limitations [28,29,32,33,34,35].

## 3. Pharmacometabolomics: The Metabolomics Approach to Practical Medicine

Pharmacometabolomic studies can elucidate drug effects and increase the ability to predict individual variation in drug–response phenotypes [26]. Using complex technologies, the field of PhM attempts to quantify drug interventions depending on a pre-dose mathematical model of an individual’s metabolic status. The so-called “metabolite profile” represents all interactions among various aspects of the genome, microbiome (the symbiotic human flora), parasites, environment, etc. Therefore, a metabolite profile is very informative not only to determine the effective dose of a given drug but also to modify pharmacotherapy on the basis of patients’ characteristics, including genetic variation, metabolism, and environmental intervention [14,19,23].

The first application of PhM in practical medicine was described by Clayton et al. [53]. The assumption was made that one can predict some aspects of drug metabolism by analyzing the metabolite profile of a patient prior to dosing. Paracetamol (acetaminophen) is often used for this purpose. This drug is widely used in medical practice; it has extremely low toxicity and well-known metabolism with rapid urinary excretion. In this study, the urine samples of 99 healthy male adult volunteers (non-smokers, without any previous drug use) were collected. The samples’ composition was analyzed pre- and post-paracetamol administration. A clear relationship between the pre-dose and post-dose metabolite profiles was observed. For several volunteers, high levels of p-cresol sulfonate (co-metabolite of microbial colonic flora) were detected in pre-dose urine. A high level of p-cresol sulfonate in pre-dose urine was correlated with a significant decrease in the concentration of paracetamol sulfonate metabolites in post-dose urine. It was concluded that p-cresol and paracetamol are competitive substrates and that the sulfonation capacity of paracetamol is inhibited by the competitive sulfonation of p-cresol. This demonstrated the necessity of drug dose correction; this approach can help to predict individual doses of other drugs for which sulfonation is an important part of catabolism (e.g., minoxidil, tamoxifen) [19]. Later, the ability of PhM to predict an individual’s drug response was also demonstrated for another class of drugs by a different research group [54,55,56]. A targeted panel of metabolites related to cholesterol metabolism was investigated. Significant correlations between gut-derived metabolites and response to simvastatin were detected. This represents further evidence that genome, microbiome, and diet all contribute to the variation in the response to simvastatin [54,55,56]. These findings indicate that assessing the effects of microbiome activity may be integral to pharmaceutical development and personalized health care. Furthermore, gut bacterial populations might be deliberately manipulated to improve drug efficacy and to reduce the frequency of adverse drug reactions.

Thus, the basic principle of PhM is finding a connection between the metabolite profile (pre-dose) and the metabolic “fate” of a drug (post-dose). Metabolic profiles could provide valuable information about treatment outcomes and could contribute to a more personalized approach to therapy.

Drug responses are often highly variable and are greatly affected by an individual’s capacity to metabolize or utilize the drug. A number of PhM studies have focused on the prediction of individual drug metabolism by measuring the cytochrome P450 activity of drug-metabolizing enzymes [57,58,59,60]. These enzymes are responsible for the metabolism of many drugs and have important effects on drug pharmacokinetics, efficacy, and safety. For example, Rahmioglu et al. used NMR-based metabolomics to identify a metabolic signature associated with a variation in induced CYP3A4 activity [25]. Pre- and postintervention fasting urine samples were used to obtain metabolite profiles. The samples were analyzed with NMR spectroscopy. Ultraperformance liquid chromatography combined with mass spectrometry was performed to obtain a marker for CYP3A4 induction: the ratio of 3-hydroxyquinine to quinine (3OH-Q/Q). In a PhM study, Diczfalusy et al. showed that the plasma concentrations of 4b-hydroxycholesterol increased after complete induction of CYP3A4/5 by carbamazepine or rifampicin. The authors suggested that 4b-hydroxycholesterol may be used as an endogenous marker of CYP3A activity [60]. Such information can help to predict CYP3A activity and the metabolism level of CYP3A-mediated drugs prior to administration. Although the field of cytochrome pharmacogenomics is now well established, the newly emerging field of PhM can be used to complement this genomic information.

An area of significant recent activity has been the use of PhM for the prediction of drug safety and efficacy [61]. Metabolic profile analysis (determination of the level of individual metabolites or groups of metabolites, as well as their combination or ratio) allows clinicians to predict the effectiveness of a drug therapy and may help clinicians to select the optimal dose and schedule of drug administration, even before use. Metabolic monitoring during treatment allows healthcare practitioners to assess the effectiveness of the selected therapy and make the necessary adjustments.

The analysis of the metabolite profile also allows clinicians to detect the biomarkers of various diseases (especially latent, undiagnosed, or poorly diagnosed diseases, before or at the beginning of therapy) [42,43]. Many of these diseases may have significant effects on drug metabolism and efficacy [62]. Information about such diseases helps to correct therapeutic interventions (e.g., replacement of drug with an analogue, variation in dosage and intervention schedule). The alterations in the levels of numerous metabolites can predict the effectiveness of a treatment or indicate the effectiveness of a therapy, making it possible to select the best medication regimen.

## 4. Comparison of Pharmacometabolomics and Pharmacogenomics

As stated above, the individualization of pharmacological treatment is the main aim of modern therapy. Initial efforts were focused on pharmacogenomics, a platform that combines pharmacology and genomics. The main concept of this approach is that genetic polymorphisms determine inter-individual fluctuations in the pharmacokinetics and pharmacodynamics of pharmacological agents [19]. Pharmacogenomic facilitates the comparison of human genomes, identifies the effects of individual genetic variation on the ability of an organism to respond to a specific medication, and predicts diseases and personalized pharmaceutical therapy (“tailoring” of therapy) according to individual genetic makeup [19].

A typical example of PhG application is the dose setting for the antithrombotic agent warfarin [63]. Dose setting is particularly important in this context, as an overdose of the drug can cause bleeding in the patient. Cytochromes CYP2C19, CYP3A4, and CYP1A2 (especially CYP2C9) play a major role in the metabolism of warfarin in the human body [64]. The presence in the patient’s genome of the alleles R144C (CYP2C9*2) and I359L (CYP2C9*3) leads to a steady decrease in cytochrome activity (up to 90% for homozygous variants) and alters the drug’s half-life [64,65]. The decrease in warfarin elimination, the increase in plasma concentration, and the development of bleeding complications are a result of allele composition. The identification of the patients with these “slow” alleles can help to correct the dose of warfarin: these patients require lower doses of the drug to achieve a similar therapeutic effect compared to patients with a wild-type haplotype [65]. Similar situations are described for a variety of other drugs: pravastatin (polymorphisms of cholesterol ester transferase) [66], neuroleptics (polymorphisms of dopamine receptor D3) [67], hydrochlorothiazide (adducin polymorphism) [68], salbutamol (polymorphism of adrenergic receptor) [69], etc. The results of these investigations have confirmed the importance of identifying the genetically determined reactions of an organism to medicine.

However, such a “direct” correlation when drug response is affected by any single genetic variation is exceptional. In most cases, the effectiveness of medication does not depend exclusively on genotype. Genetic modifications play a limited role: they can increase the possibility of response to the drug and the development of ADR. The actual response of an organism to medication is determined by complex interactions between an individual’s genotype and non-genomic factors. The effect of non-genomic factors on the phenotype is significant. Frequently, these factors can modulate the individual phenotype, leading to the appearance of inter-individual differences in response to the drug [70].

One example of the modifying influence of external factors is the effect of grapefruit juice components on the expression of cytochrome P-450 (CYP3A4) and, accordingly, on the pharmacokinetics of CYP3A-mediated drugs [71]. Addictions (tobacco smoking, alcoholism, etc.), environmental factors (lifestyle, diet, stress, etc.) can also modulate the effect of drug therapy (by both pharmacokinetic and pharmacodynamic mechanisms) [72]. Genetic variability among ethnic groups and the polygenic nature of the organism’s response to a drug can prevent the correct prediction of drug effectiveness by PhG [73].

All these examples demonstrate the difficulties related to the interpretation and the limitations of pharmacogenomics tests: in most cases, the approach cannot predict the response to medication with 100% accuracy. Thus, the PhG approach can be applied only for a limited number of drugs. An application of the approach is correct if a direct relationship between genetic variation, drug response, and phenotype is proven. In recent years, several genetic variations were found to predict a drug response with precision [74]. This fact led to some disappointment with the wide clinical application of pharmacogenomics tests. The inability to monitor the disease state and the efficacy of a current treatment, as well as the limitations of predicting the effectiveness and toxicity of drug administration render PhG insufficient for a personal approach to treatment. Furthermore, there is still a lack of clinical evidence from randomized controlled trials demonstrating this benefit with statistical significance. Pharmacogenomics testing is not currently recommended in the clinical practice [61].

In contrast to genes and genetic risk scores, which can be used to indicate what might happen, metabolic profiling and metabolic phenotyping indicate what is currently taking place [75] (Figure 2). Metabolites represent both the downstream output of the genome and the upstream input from the environment. Therefore, the study of metabolites and metabolism (metabolomics) allows scientists to explore gene–environment interactions. The concept of PhM is that some individuals will respond differently to drug administration because their differentiated pre-dose phenotypes predispose them to do so. Pre-dose phenotypes are influenced by the subjects’ genomes and by their environment, as well as by the status of their microbiome and its interaction with their genome.

## 5. Comparison of Pharmacometabolomics and Pharmacoproteomics

Another approach to the personalization of treatment based on “omics” technologies is a branch of proteomics denoted as PhP. Pharmacoproteomics focuses on the identification and quantification of biosamples’ protein content under various pathophysiological conditions (including disease), changes in protein expression, and possible modifications to a protein before and after drug therapy.

Pharmacoproteomics provides a more functional representation of patient-to-patient variation than that provided by genotyping. Because it includes the effects of posttranslational modification, PhP connects the genotype with the phenotype—a connection that is not always predicted by genotyping alone. For example, a silent single nucleotide polymorphism can give rise to two or more variant forms of mRNAs that do not produce an altered amino acid sequence in the encoded proteins but can alter a phenotype by inducing changes in mRNA folding. An understanding of mRNA conformational changes could lead to new drug targets, such as allele-specific targets [76].

The proteomics-based characterization of multifactorial diseases may help to match a particular target-based therapy to a particular biomarker in a subgroup of patients. The commercial pharmaceutical sector is taking a lead in developing this area. Individualized therapy may be based on differential protein expression rather than genetic polymorphisms [76].

At the present time, the most promising application of PhP appears to involve the field of oncology. The approach is used to identify tumor-specific protein markers and to evaluate the action of anti-cancer drugs [77,78]. The “popularity” of the field of PhP, which is guided to the prediction of drug dosing, remains low [79]. There are limitations of applying PhP as a method for personalizing treatment: the difficulty of sample preparation, unstable sample protein content (e.g., proteins degrade intensively at room temperature, low temperature reduces proteolytic activity, destroys erythrocytes, and releases intra-erythrocytic proteins), etc. All these factors lead to the difficulty of interpreting proteomic profiles with the aim of predicting the effectiveness of a medication.

The main advantage of PhM over PhP, as well as PhG, is the application of a metabolite profile for analysis. In spite of the fact that the metabolome is exposed to fluctuations in time (due to environmental factors, etc.), the general metabolome remains invariant for each individual [80]. The ability of the metabolome to provide complete information about the current physiological status of a patient makes metabolic profiling most suitable for the personalization of treatment. The approach can be used to identify biomarkers, which, in turn, allow clinicians to monitor disease status and ADR, as well as to evaluate drug toxicity and drug efficacy. Based on this information, appropriate clinical treatment can be defined. Some investigations have been devoted to study the application of this method for the treatment of autoimmune diseases [81], cardiovascular disease [82], and inflammatory bowel disease [83].

## 6. Comparison of Pharmacometabolomics and Therapeutic Drug Monitoring

The most common method used in the clinic for the personalization of drug treatment is TDM [21]. Therapeutic drug monitoring is close to targeted metabolomics: the measurement of defined, chemically characterized, and biochemically annotated substances. Therapeutic drug monitoring can be defined as a method for the quantitative determination of drug concentration in various biofluids, requiring the construction of calibration curves. This approach aims to find the individual dosage to support optimal drug concentration in biofluids (maximal effectiveness and minimal toxicity) [84]. Therapeutic drug monitoring is based on the existence of a direct connection between the administered dose of a drug (biofluid drug concentration) and the drug’s therapeutic effects [85]. The use of TDM for the individual optimization of drug therapy has become widespread over the last decade. For example, TDM of antipsychotic drugs is the specific method of clinical pharmacology, which involves measurement of drug serum concentrations followed by interpretation and good cooperation with the clinician [86]. Sometimes, the application of this approach is a necessary and obligatory condition for therapy, for instance, when treatment involves mediation with a narrow range of effective concentrations (aminoglycosides, tacrolimus, methotrexate, lithium) or a strong individual variation in drug pharmacokinetics (induced by age, body mass index) [84].

However, TDM has several significant limitations as a method for the personalization of treatment. Firstly, the approach cannot predict drug effectiveness and toxicity. Secondly, the approach records the concentration of drug that reached its target, i.e., pharmacokinetics only. Thus, the application of TDM is correct for groups of drugs with a “direct” relationship between concentration in the biofluids of an individual and individual response. The approach disregards the contribution of inter-individual pharmacodynamic variability (e.g., influence of drugs on the target, signaling downstream of the target) [87]. Numerous factors can alter drug pharmacodynamics: genetic factors, drug interaction, disease (hepatic, renal), pregnancy, etc. [88]. In the presence of these factors, the dosage suggested by TDM may be incorrect, and additional dosage adjustment may be required.

In other words, TDM may be useful for establishing the initial dosing regimen and monitoring certain medications. However, TDM cannot compensate for errors in diagnosis, poor drug choice, errors in dispensing and dosage, sampling errors, etc.

Unlike TDM, PhM (an application of untargeted metabolomics) makes conclusions based on the analysis of the metabolite profile, which is intended to serve as a comprehensive analysis of all the measurable metabolites in a sample, including unknown chemicals. The existing levels of metabolites (the final products of cellular processes) are the ultimate response to these factors, including drug administration [89]. This use of the metabolite profile allows the clinician to monitor drug levels and levels of drug metabolites (pharmacokinetic index) and to monitor metabolite levels, as an index of the organism’s response to drug administration, thus permitting indirect estimates of the drug’s pharmacodynamic index. Thus, the application of PhM increases the chance of selecting the optimal treatment, allowing clinicians to predict and monitor a drug’s effectiveness.

## 7. Conclusions

In the present postgenomics era, the significance of metabolomics as one of the latest ‘‘omics’’ platforms for assessing drug effectiveness and toxicity has increased tremendously. The utility of metabolomics in medical initiatives is not surprising. As noted above, genes and genetic risk scores can be used to indicate what might happen in terms of biochemical or cellular functions, whereas metabolic profiling and metabolic phenotyping indicate what is happening at a biochemical level. Metabolic profiling, which can simultaneously identify thousands of metabolites, has shown significant results in many scientific and clinical applications to date.

Pharmacometabolomics is a new approach based on the practical application of metabolite profiles of easily accessible biofluids. The PhM approach can be used to predict the effectiveness of a drug prior to dosing and to monitor the post-dose effectiveness of the medication and the disease development, thus avoiding ADR. Pharmacometabolomics is a personal approach to therapy that achieves the goal of personalized medicine: “the right drug for the right patient at the right dose”. One of the main problems of implementing PhM in clinical practice is the difficulty of standardizing the metabolomics methods. However, some methods, including DIMS, can be considered as suitable for standardization to the level required for medical purposes.

## Figures and Tables

**Table 1:** Metabolomics analytical techniques for personalized medicine.

Techniques		Advantages	Disadvantages	References
Nuclear magnetic resonance spectroscopy (NMR)		- Does not require prior separation or chemical derivatization of metabolites - High reproducibility - All metabolites can be simultaneously detected - Determination of the concentration of metabolites - The sample remains unchanged, it can be used for further analysis	Low sensitivity, only several tens of metabolites with a relatively high concentration can be detected	Robertson et al. [27] Lindon et al. 2003 [28] Lindon et al. 2005 [29]
Mass spectrometry (MS)	Gas chromatography–MS (GC–MS)	- High sensitivity, hundreds or thousands of metabolites can be detected - Ability to distinguish isomers of metabolites - More suitable for identification: there are many EI (electron impact)–MS libraries for the identification of analyte-based GC–MS data	- Lengthy analyses times - Requires a prior separation of the different isomers of metabolites - Derivatization is required to increase the volatility and thermal stability of the drugs that are nonvolatile, polar, or thermally labile	German et al. [35]
	Liquid chromatography–MS (LC–MS)	- High sensitivity, hundreds or thousands of metabolites can be detected - Ability to distinguish isomers of metabolites - Useful for non-volatile compounds	- Lengthy analyses times - Requires a prior separation of the different isomers of metabolites - Unable to use EI and EI–MS libraries	Liu et al. [30] Chen et al. [31] Kristal et al. [32] Beal et al. [33] Ogawa et al. [34]
	Direct-infusion mass spectrometry (DIMS)	- Fast and highly reproducible - Without any preliminary separation - Requires a small amount of sample	- Suppression of the signal of individual metabolites (ion suppression), - Interference of mass peaks from different metabolites - Impossibility to distinguish isomers of metabolites	Musharraf et al. [36] Lokhov et al. [37] Drexler et al. [38]
